# Impending relapse of myelodysplastic syndrome after allogeneic transplant is difficult to diagnose and requires a multi-modal approach

**DOI:** 10.1186/s12907-017-0066-8

**Published:** 2017-12-28

**Authors:** Elizabeth L. Courville, Megan Griffith, Celalettin Ustun, Sophia Yohe, Erica Warlick

**Affiliations:** 1 Department of Laboratory Medicine and Pathology, University of Minnesota, 420 Delaware St SE, MMC 609, Minneapolis, MN 55455 USA; 2 Division of Hematology, Oncology, and Transplantation, Department of Medicine, University of Minnesota, Minneapolis, MN USA

**Keywords:** Allogeneic stem cell transplant, Myelodysplastic syndrome, Relapse, Cytogenetic, Acute myeloid leukemia

## Abstract

**Background:**

The only potentially curative therapy for myelodysplastic syndrome is allogeneic hematopoietic cell transplant; unfortunately, there is a high relapse rate. The objective of this study was to perform a detailed clinicopathologic study of patients with relapsed myeloid neoplasm following allogeneic hematopoietic cell transplant for myelodysplastic syndrome.

**Methods:**

Pre-transplant, post-transplant, and relapse bone marrow and peripheral blood morphologic features (including dysplasia) were retrospectively evaluated by study authors. Clinical features and results of cytogenetic analysis and engraftment/chimerism studies were obtained from the medical record.

**Results:**

Our study describes 21 patients with a median time to relapse of 6 months (range 2–82). Ten of the patients relapsed with higher grade disease, including six with overt acute myeloid leukemia. Pre-transplant megakaryocyte dysplasia was associated with dysplastic megakaryocytes in the relapse specimen; however, neither erythroid dysplasia nor granulocytic dysplasia were associated with their counterpart in the relapse specimen. Relapse specimens had a lower marrow cellularity and higher blast percentage than pre-transplant disease. Cytogenetic comparisons before and after transplant showed variety, including clonal evolution (22%), the same abnormal clone (33%), or a different abnormal clone (22%).

**Conclusions:**

Our detailed review of post-transplant marrow biopsies prior to relapse highlights the difficulty in diagnosing relapse and particularly impending relapse.

## Background

Myelodysplastic syndromes (MDS) are the most commonly diagnosed myeloid neoplasms in the United States. MDS are clonal hematopoietic neoplasms characterized by ineffective hematopoiesis and varying risk of transformation to acute myeloid leukemia (AML). According to the World Health Organization (WHO) [1], MDS are further subclassified based on the number of dysplastic lineages, the number of cytopenic lineages, the percentage of ring sideroblasts, the bone marrow and peripheral blood blast percentages, the presence or absence of Auer rods, and cytogenetic findings. This heterogeneous group has variable prognoses and treatments ranging from supportive care only to chemotherapy (hypomethylating agent based therapy or intensive AML-type induction chemotherapy) to possible subsequent allogeneic hematopoietic cell transplant (HCT) [2, 3]. Therapy choice is guided by risk stratification based on the International Prognostic Scoring System (IPSS) and revised IPSS (IPSS-R) [4, 5], as well as other patient factors including age, performance status, transfusion needs, and response to first-line therapy, and donor options.

The only potentially curative therapy for MDS is allogeneic HCT. Unfortunately, relapse remains a concern with rates in the 20–50% range [6–8]. The relapse risk likely depends on multiple factors including the preparative regimen (myeloablative versus non-myeloablative or reduced-intensity), stem cell and donor source (umbilical cord blood versus sibling or adult unrelated bone marrow/peripheral blood), and pre-transplant MDS disease burden as well as MDS disease characteristics. The optimal method or combination of methods to detect impending relapse following transplant is not clear nor is the optimal therapeutic intervention for impending relapse [7].

In this study, we performed a detailed assessment of 21 patients with relapsed myeloid neoplasm following allogeneic HCT. We evaluated diagnostic MDS characteristics (IPSS, WHO classification, cytogenetics) as well as a comparison of pre-transplant and relapsed disease morphology, cytogenetics, and flow cytometry. Intervening (post-transplant, pre-relapse) data was also reviewed. Late relapse cases arising >6 months after transplant were compared to early relapse cases.

## Methods

This retrospective study was approved by the University of Minnesota Institutional Review Board (Study Number 1312 M46725) and performed according to the ethical standards of our institution. As part of the approval process by the University of Minnesota IRB, it was determined that informed consent was not required for this retrospective research study. Patients were identified by search of the bone marrow transplant database. Adult patients (≥18 years old) were included if they received their first allogeneic HCT for MDS at the University of Minnesota between 2000 and 2015. Patients were excluded if their pre-transplant bone marrow biopsy slides were not reviewed at our institution, if no post-transplant biopsy was obtained, or if there was persistent marrow disease post-transplant. The electronic medical record was used to extract clinical information. Pathology reports from the following specimens were reviewed for all patients: [1] original MDS diagnosis, [2] pre-transplant MDS diagnosis, [3] immediate pre-transplant biopsy (following cytoreductive therapy), [4] post-transplant biopsy specimens interpreted at our institution up to and including [5] the post-transplant relapse biopsy. The information extracted included the bone marrow cellularity and blast percentage, peripheral blood counts, and circulating blast percentages. The post-transplant cases, [4], were categorized as “negative” or “indeterminate” for morphologic evidence of myeloid neoplasm based on review of the original pathology report.

Available slides from the pre-transplant MDS diagnosis [2] (slides available for 15 patients) and post-transplant relapse marrow [5] (slides available for 20 patients) were reviewed by study author EC and scored for dysplasia in a semi-quantitative manner modified from the system used by Weinberg, et al. [9]. Reviewed slides included H&E stained slides of the trephine core, immunohistochemical stains performed at the time of diagnosis, Wright-Giemsa stained marrow aspirate and peripheral blood slides, and Dacie (iron) stained marrow slides. Dysplasia was scored in each lineage in increments of 10%. Specific dysplastic features in the megakaryocyte lineage were: micromegakaryocytes, hypolobated or monolobated megakaryocytes of normal size, megakaryocytes with two or more separated rounded nuclear lobes. Specific dysplastic features in the erythroid lineage were: megaloblastoid change, multinucleation, nuclear irregularities, pyknosis, and basophilic stippling. Ring sideroblast percentage was documented. Specific dysplastic features in the granulocyte lineage were: abnormal nuclear shape and hypogranulation. A lineage was only evaluated if sufficient cells were available for analysis.

To evaluate for morphologic features of impending relapse, slides from the bone marrow biopsy specimen immediately prior to the relapse specimen were reviewed by study author SY, who was blinded to the original pathology interpretation and the results of corresponding ancillary studies. The presence or absence of dysplasia, and affected lineages, was documented, and blasts were evaluated as increased or decreased. Based on the morphology alone, the specimens were re-interpreted as “negative”, “positive”, or “indeterminate” for relapsed myeloid neoplasm.

Flow cytometric studies and cytogenetic analysis were performed using standard techniques at the time of diagnosis, with the interpretive reports reviewed for this study. Flow cytometry studies evaluated for aberrant antigen expression on myeloid blasts using either 4-color or 10-color panels. Antigens evaluated included CD3, CD7, CD10, CD13, CD14, CD15, CD19, CD33, CD34, CD45, CD56, CD117, and HLA-DR. Myeloid maturation patterns by flow cytometry were not evaluated. Engraftment/chimerism analysis was performed as previously described [10], with interpretive reports reviewed for this study.

Statistical analysis was performed using the IBM SPSS Statistics program version 22. A two-tailed Fisher’s exact test was used for categorical data and a Mann-Whitney U test (for independent samples) or Wilcoxon signed-ranks test (for related samples) was used for continuous data.

## Results

Our patient cohort included 10 males and 11 females. The median age at transplant was 59 years (range 34 to 71). Three patients had a medication history compatible with therapy-related myeloid neoplasm. One patient received cyclophosphamide for scleroderma/interstitial lung disease for 2 years ending 3 years prior to MDS diagnosis, one patient received multi-agent chemotherapy for breast cancer (including paclitaxel, doxorubicin, and cyclophosphamide) 4 years prior to MDS diagnosis, and one patient was treated with doxorubicin and cyclophosphamide for breast cancer 10 years prior to MDS diagnosis.

Eighteen patients were treated with a non-myeloablative/reduced intensity conditioning regimen prior to transplant (cytarabine, fludarabine, and total body irradiation with or without anti-thymocyte globulin, *n* = 17, or busulfan/fludarabine, *n* = 1) and three were treated with a myeloablative conditioning regimen (busulfan and cytarabine or cytarabine and fractionated total body irradiation). Graft-versus-host-disease prophylaxis included cyclosporine and mycophenolate mofetil (*n* = 17), cyclosporine and methotrexate (*n* = 3) or methotrexate and tacrolimus (*n* = 1). Source of donor stem cells included umbilical cord blood (*n* = 10), sibling (*n* = 9), and unrelated donor (*n* = 2).

### Pre-transplant MDS versus relapse characteristics

Patient were most commonly transplanted for refractory anemia with excess blasts (RAEB-1 or 2, *n* = 13) and refractory cytopenias with multilineage dysplasia (RCMD, *n* = 3). In eight patients, disease prior to transplant included ring sideroblasts, with five having a ring sideroblast percentage ≥ 15%. Of the relapses, six cases relapsed with overt AML and one additional case (Case 75) progressed to AML 2 months after initial relapse (only withdrawal of immunosuppression prior to progression). Two cases relapsed as MDS with increased blasts where the pre-transplant disease lacked excess blasts, and an additional case lacked an increase in blasts in both the pre-transplant and relapse specimens but progressed to RAEB-1 2 months after initial relapse despite a reduction in immunosuppression in the intervening time.

The presence of dysplastic megakaryocytes in the pre-transplant specimen showed a statistically significant association with the presence of dysplastic megakaryocytes in the relapse specimen (*p* = 0.018) with increased significance (*p* = 0.001) when a dysplastic megakaryocyte threshold of 50% was applied. In contrast, neither erythroid dysplasia/ring sideroblasts in the pre-transplant specimen nor granulocytic dysplasia in the pre-transplant specimen was associated with their counterpart in the relapse specimen (*p* = 0.520/1.0, and 0.070, respectively).

There was a significant difference between the pre-transplant and relapse bone marrow cellularity [pre-transplant median of 73% (range 15–95%) and relapse median of 45% (range 10–90%), *p* = 0.003] and blast percentage [pre-transplant median of 5% (range 1–19%) and relapse median of 10% (range 0.2–51%), *p* = 0.023] but there was no significant difference between the pre-transplant and relapse peripheral blood blast percentage. There was a significant difference between the pre-transplant and relapse platelet count [pre-transplant median of 73 × 10^9^/L (range 5–1242) and relapse median 38 × 10^9^/L (range 7–189), *p* = 0.028] and a borderline significant difference between the pre-transplant and relapse white blood cell count [pre-transplant median 3.3 × 10^9^/L (1–33 range) and relapse median 2.5 × 10^9^/L (0.6–7.6 range) *p* = 0.054]. No statistically significant difference was seen between the pre-transplant and relapse hemoglobin, mean corpuscular volume (MCV) or absolute neutrophil count.

The majority (18/21, 86%) of cases had an abnormal karyotype in the myeloid neoplasm before and/or after transplant. Pre- and post-transplant cytogenetic comparisons were categorized as follows: same abnormal relapse clone (6/18, 33%), relapse clone with some similarities (4, 22%), relapse clone showing clonal evolution (4, 22%), and different relapse clone (4, 22%). Some cases were placed in the “relapse clone with some similarities” category because only targeted FISH analysis and not a full karyotype was performed at the time of relapse.

Flow cytometry data was available for a subset of specimens. Of the nine pre-transplant flow cytometry studies, three showed an increase in blasts and three showed an abnormal immunophenotype on blasts (heterogeneous/partial CD7 expression or homogenous expression of antigens/discrete cluster). The majority of relapse disease flow cytometry specimens (15/19, 79%) had increased blasts. In those cases without increased blasts, immunophenotypic abnormalities noted but not considered definitive included heterogenous or partial CD7 expression or homogenous expression of myeloid markers such as CD13 and CD33. A single pair of pre-transplant and relapse flow cytometry studies showed immunophenotypic similarities with partial CD7 expression in both.

### Early versus late relapse (Table 1)

The median time to relapse after transplant was 6 months (range 2–82). Eleven patients had a late relapse, defined as >180 days (6 months) after transplant, with a median time to relapse of 15.4 months (range 6–81.6 months). The ten patients with early relapse had a median time to relapse of 3 months (range 2.2–5.8). Table 1 compares the patients with early versus late relapse.

**Table 1:** Comparison of Hematologic and Morphologic Features Between Early and Late Relapse Patients

	Early Relapse (<6 months) *n* = 10	Late Relapse (>6 months) *n* = 11	*p*-value
Age at transplant, years, median (range)	67 (34–71)	58 (46–61)	**0.013**
Gender, M:F	5:5	6:5	NS
Time to relapse from transplant, months, median (range)	3 (2–6)	15 (6–82)	**<0.001**
Pre-transplant MDS specimen, peripheral blood and bone marrow characteristics			
Hgb, g/dL, median(range)	9.0 (7.6–12.7)	9.3 (5.6–12.9)	NS
MCV, fL, median (range)	89 (73–109)	101 (86–107)	**0.023**
WBC, × 10^9^/L, median (range)	3.0 (1.1–10.2)	3.3 (1–33)	NS
ANC, × 10^9^/L, median (range)	0.9 (0.1–7.8)	1.1 (0.4–24.1)	NS
Platelets, × 10^9^/L, median (range)	36 (10–1242)	89 (5–206)	NS
Blood blasts, %, median (range)	1 (0–16)	0.5 (0–13)	NS
Dysgranulopoiesis^a^	4/7	3/8	NS
Dyserythropoeisis^a^	5/7	6/8	NS
Dysmegakaryopoiesis^a^	4/6	5/3	NS
Ring sideroblasts^a^	4/8	4/9	NS
Marrow cellularity, %, median (range)	90 (15–95)	43 (30–95)	0.075
Bone marrow blasts, %, median (range)	4 (1–19)	7 (2–14)	NS
Increased marrow blasts (>5%)	7/10 (70%)	8/11 (72%)	NS
Relapse myeloid neoplasm specimen, peripheral blood and bone marrow characteristics			
Hgb, g/dL, median(range)	10.0 (8.6–12.2)	9.8 (8.1–13)	NS
MCV, fL, median (range)	88 (80–105)	99 (87–113)	0.051
WBC, × 10^9^/L, median (range)	2.2 (0.6–7.6)	2.5 (1.5–3.4)	NS
ANC, × 10^9^/L, median (range)	1.3 (0.2–5.9)	1.2 (0.2–2.5)	NS
Platelets, × 10^9^/L, median (range)	20 (7–128)	66 (10–189)	0.061
Blood blasts, %, median (range)	0.3 (0–15)	2 (0–38)	NS
Dysgranulopoiesis^a^	3/9	2/11	NS
Dyserythropoeisis^a^	5/9	6/11	NS
Dysmegakaryopoiesis^a^	7/8:5/8	6/10:3/10	NS
Ring sideroblasts^a^	6/6	1/3	**0.033**
Marrow cellularity, %, median (range)	48 (20–90)	45 (10–90)	NS
Bone marrow blasts, %, median (range)	5 (0.2–30)	14 (4–51)	**0.029**
Increased marrow blasts (>5%)	5/10 (50%)	10/11 (91%)	0.063

Bold indicates statistical significance

^a^Any degree of dysplasia ≥10% of a lineage. See text (materials and methods section) for specific dysplastic features included for this study; “n” for dysplasia and ring sideroblast evaluation varies for each category depending on the slides and number of precursors in each lineage available for review

To summarize, patients with late relapse had a younger median age at transplant and had a higher median bone marrow blast percentage at relapse. Donor source (umbilical cord blood versus non-umbilical cord blood), conditioning regimen (myeloablative versus non-myeloablative), and IPSS risk score were not associated with timing of relapse, although the number of patients in each category was small. All three therapy-related MDS cases were late relapses, occurring 6, 19, and 49 months after transplant. Dysplasia characteristics in the pre-transplant or relapse specimens did not show a statistically significant association with timing of relapse, nor did the cytogenetic comparison. There was no association between relapse as overt AML (>20% blasts) and timing of relapse.

### Pre-relapse marrow evaluation

Post-transplant bone marrow biopsy specimens evaluated at our institution up to and including the relapse specimen are presented in Tables 2 and 3 (divided into early and late relapse cases) including morphologic interpretation, engraftment results, and cytogenetics results. There was no significant association between a morphologic interpretation of indeterminate and subsequent morphologic interpretation of relapse (6 of 15 specimens interpreted as indeterminate and 14 of 48 specimens interpreted as negative had morphologic relapse in the subsequent marrow biopsy, *p* = 0.528).

**Table 2:** Early Relapse Cases

Study No	Age at transplant/IPSS score	Original MDS Diagnosis	Pre-transplant MDS Diagnosis	Immediate pre-transplant Marrow Biopsy (following cytoreductive therapy)	Post-transplant Assessment 1	Post-transplant Assessment 2	Post-transplant Assessment 3	Post-transplant Assessment 4	Follow-up	Cytogenetics Comparison
		Diagnosis/CG	Diagnosis/CG	Diagnosis/CG	Morphologic Conclusion/Engraftment^a^/CG	Morphologic Conclusion/Engraftment^a^/CG	Morphologic Conclusion/Engraftment^a^/CG	Morphologic Conclusion/Engraftment^a^/CG	Status/Relapse Treatment	
7	61/INT-1	RARS /46,XX,del(12)(p11.2p13) [7]/46,XX [13]	RARS/46,XX,del(12)(p11.2p13) [3]/46,XX [17]	No intervening marrows pre-transplant	Indeterminate /6 /Neg by FISH	**MDS with dyerythropoiesis and numerous ring sideroblasts, no increase in blasts** ^**c**^ **/88 /46,XX[49]//46,XY[1]**			Deceased of invasive aspergillus lung infection with persistent MDS/ reduction in immunosuppression then DLI for subsequent RAEB-1	Some similarities between clones
Days^b^		−699	−15		23	100			237/137 (post-relapse)	
27	69/Unknown	RCMD-RS	RCMD-RS/45,XY,-7,del(20q)(q11.2q13.1) [3]	No intervening marrows pre-transplant	Indeterminate /30.6 / monosomy 7 by FISH (8% interphase cells)	Negative /0 /monosomy 7 by FISH (2.75%, slightly above normal control range)	Negative /0 /not performed	**MDS with dysmegakaryopoiesis, numerous ring sideroblasts, no increase in blasts /12.6 /45,XY,-7,del(20q)(q11.2q13.3)[3]/46,XY[12]**	Unknown/ Unknown	Same abnormal clone
Days		−111	−31		22	60	98	155		
32	62/INT-1	RAEB-1	RAEB-2/clone with trisomy 8	Hypercellular marrow withno increase in blasts; 3.25% by FISH with extra chromosome 8	Indeterminate /6.1 /Neg by FISH	Indeterminate /11.8 /Neg by FISH and karyotype	**AML /41.8 /46,XX,t(2;3)(q23;q26–27)[4]/46,XX[14]**		Unknown/ Unknown	Different abnormal clone
Days		−376	−141	−29	33	63	98			
34	49/HIGH	RAEB-1 /complex karyotype including deletion of 5q	Same specimen as original MDS diagnosis	Normocellular marrow with no increase in blasts/cytogenetics not performed	Negative /57.4 /Neg by FISH	Negative /29.6 /loss of chromosome 5 by FISH (6.75% interphase cells) and complex karyotype	**MDS with dysgranulopoiesis, borderline increased marrow blasts, and rare circulating blasts / 20.3/FISH: 19.5% had a signal pattern indicative of loss of both 5p15.2 and q31**		Unknown/ Unknown	Some similarities between clones
Days		−200		−25	21	36	65			
58	67/LOW	MDS with isolated del(5q) /46,XY,del(5)(q13q33) [18]/46,XY [2]	RAEB-2/46,XY,del(5)(q22q33) [18]/46,XY [2]	No intervening marrows pre-transplant	Indeterminate /0 /Neg by FISH	Indeterminate /3 /Neg by FISH	RAEB-1 /38 /46,XY,del(5)(q22q35)[11*]/46,XY[7]/46,XX[2]		Unknown/ Unknown	Same abnormal clone
Days		−458	−25		21	98	137			
63	68/INT-2	RCMD-RS /45~46,XY,add(4)(q31),del(5)(q15q33),del(7)(q22q34),+8,del(11)(q14q23),add(12)(p11.2),dic(14;15)P(11.2;p11.2),add(17)(p11.2),-18,01mar[cp18]/46,XY [2]	RAEB-2/44–46,XY,-3,del(4)(q31q35),del(5)(q15q33),del(7)(q22q36),+8,del(11)(q21q23),add(12)(p11.2),dic(14;15)(p11.2;p11.2),-17,-18,+2mar[cp5]	Normocellular marrow with no increase in blasts/ Negative by FISH	Negative /2.9 /Neg by FISH	**RAEB-2 /91.8 /FISH: 83% had a signal pattern consistent with deletion of the long arm of one chromosome 5**			Unknown/ azacitidine	Some similarities between clones
Days		−298	−126	−15	21	90			129/39 (post-relapse)	
78	69/LOW	RARS-T /46,XY,dup(1)(q21q43) [14]/46,XY,der(15)t(1;15)(q12;p11.2) [3]/46,XY [3]	Same specimen as original MDS diagnosis ^d^	No intervening marrows pre-transplant	Indeterminate/0 /Neg by FISH	Negative/7 /Neg by FISH	Abnormal with 5% ring sideroblasts /14 /46,XY,dup(1)(q21q43)[2]/46,XY,der(15)t(1;15)(q12;p11.2)[1*]/46,XY[17]		Deceased with steroid refractory late acute GVHD/No intervention	Same abnormal clone
Days		−21			23	100	174		232/58 (post-relapse)	
79	70/INT-2	RAEB-1 /43–44, XX, −5, −7, −19, −22, +12mar [3]/4245, sl, −18, +14[11]/46,XX [6]	Same specimen as original MDS diagnosis	Persistent MDS with 3% marrow blasts/44,XX,-5,-7,-18,-19,-22,+3mar [2]/46,XX [19]	Negative/ not performed /Neg by FISH	**Suspicious for myeloid neoplasm with slight trilineage dyspoiesis and 4% to 5% blasts /23 /44,XX,-5,-7,-19,-22,+mar1,+mar2[3]/43,idem,-18[2]//46,XY[15]**			Alive currently undergoing treatment for relapse (as AML) that occurred 2 years after DLI/ immunosuppression withdrawal	Same abnormal clone
Days		−70		−28	20	90			625/535 (post-relapse)	
81	71/INT-1	RAEB-1	RAEB-1/Normal karyotype	No evidence of residual disease/cytogenetics not performed	Negative^e^ /38 /normal karyotype	**RAEB-1 / 26/46,XX[19]**			Deceased with persistent disease/withdrawal of immunosuppression then ALT-803 trial	No clonal abnormality pre-transplant disease and relapse
Days		−735	−165	−38	21	98			465/367 (post-relapse)	
82^f^	34/INT-1	RCMD /46,XY,add(6)(?p21.2) [18]/46,XY [2]	RAEB-1/46,XY,add(6)(p21.1) [20]	No intervening marrows pre-transplant	Indeterminate/0 /normal karyotype	Indeterminate /0 /normal karyotype	Indeterminate /1 /not performed	**AML /66 /46,XY,add(6)(p21.1)[4]/46,idem,t(9;14)(q34;q24)[14]/46,X,t(Y;1)(p11.3;q21),del(4)(q11), der(13)t(4;13)(q11;p13),add(6)[2]**	Deceased with persistent disease/ 7 + 3 induction, ALT-803, GCLACchemotherapy, azacitidine, DLI	Clonal evolution
Days		−230	−35		21	62	99	139	374/235 (post-relapse)	

RELAPSE MARROWS in BOLD

*Abbreviations*: *IPSS* International prognostic scoring system, *MDS* myelodysplastic syndrome, *CG* cytogenetics, *INT-1* Intermediate-1, *INT-2* Intermediate-2, *RARS* Refractory anemia with ring sideroblasts, *RCMD* refractory cytopenias with multilineage dysplasia, *RCMD-RS* refractory cytopenias with multilineage dysplasia and ring sideroblasts, *RAEB* refractory anemia with excess blasts, *AML* acute myeloid leukemia, *FISH* fluorescence in-situ hybridization, *DLI* donor lymphocyte infusion, *GCLAC* G-CSF priming, clorarabine, and high dose cytarabine, *ALT-803 trial* IL-15 Superagonist Clinical Trial, *GVHD* graft versus host disease

^a^Engraftment is reported as % recipient

^b^Days are relative to transplant date unless otherwise noted

^c^Bone marrow biopsy 2 months after showed RAEB-1

^d^Patient carries diagnosis of RARS-T since 2007, reason for transplant is clonal progression and development of transfusion requirements

^e^Case 81 was called positive on study review due to increased blasts

^f^ Received myeloablative chemotherapy pre-transplant

**Table 3:** Late Relapse Cases

Study No	Age at transplant/IPSS score	Original MDS Diagnosis	Pre-transplant MDS Diagnosis	Immediate pre-transplant Marrow Biopsy (following cytoreductive therapy)	Post-transplant Assessment 1	Post-transplant Assessment 2	Post-transplant Assessment 3	Post-transplant Assessment 4	Post-transplant Assessment 5	Post-transplant Assessment 6	Post-transplant Assessment 7	Post-transplant Assessment 8	Post-transplant Assessment 9	Follow-up	Cytogenetics Comparison
		Diagnosis/CG	Diagnosis/CG	Diagnosis/CG	Morphologic Conclusion/Engraftment^a^/CG	Morphologic Conclusion/Engraftment^a^/CG	Morphologic Conclusion/Engraftment^a^/CG	Morphologic Conclusion/Engraftment^a^/CG	Morphologic Conclusion/Engraftment^a^/CG	Morphologic Conclusion/Engraftment^a^/CG	Morphologic Conclusion/Engraftment^a^/CG	Morphologic Conclusion/Engraftment^a^/CG	Morphologic Conclusion/Engraftment^a^/CG	Status/Relapse Treatment	
10^b,c^	51 /INT-2	RCMD/45,XX,-5,add(7)(q11.2) [8]/41–43,XX,-5,add(7)(q11.2),-12,-16,-18,-20[cp4]/46,XX [9]	RCMD/45,XX,-5,add(7)(q11.2) [4]/42,idem,-12,-16, −20[6]/49,XX [10]	No intervening marrows pre-transplant	Negative /0 /Neg by karyotype	**AML /47.6 /Abn by FISH. 36% with loss of the long arm of one chromosome 5 and 41.5% had a signal pattern consistent with loss of the long arm of chromosome 7**								Deceased of sepsis with persistent disease/ 7 + 3 induction, LYF73636, Haplo NK	Some similarities between clones
Days^d^		−129	−17		95	1477								1599 /122 (post-relapse)	
17	50 /INT-1	RCMD/ 46,XX,+1,der(1;7)(q10:p10) [15]/46,XX [5]	RCMD/46,XX,+1,der(1;7)(q10;p10) [5]/46,XX,-7,+21[11]/46,XX [4]	No intervening marrows pre-transplant	Negative /0 /Neg by FISH	Negative /0 /not performed	Negative /0 /not performed	Negative /0 /not performed	Negative /0 /Neg by FISH	Negative /0 /Neg by FISH	**RAEB-2 /15 /45,XX,-7[4]//46,XY[108]**			Deceased of GVHD, stroke, and multiorgan dysfunction with persistent disease/ Chemo + DLI	Some similarities between clones
Days		−849	−17		21	61	98	182	367	733	2449			2604 /155 (post-relapse)	
33	46 /INT-2	RAEB-2/46,XX [20]	Same specimen as original MDS diagnosis	Normocellular marrow with no dysplasia and no increase in blasts/normal karyotype	Negative /37.5 /not performed	Negative /0 /not performed	Negative /0 /not performed	Indeterminate ^e^ /0 /46,XX,t(6;12)(p22;p13) [3]/46,XX [1]//46,XX[16]	**AML /26.1 /46,XX,t(6;12)(p22;p13)[9]//46,XX[11]**					Alive in on-going remission/ Induction Chemotherapy then Haplo NK with transplant	Different abnormal clone
Days		−90		−17	21	101	182	364	410					2302 /1892 (post-relapse)	
39	59 /INT-1	RAEB-1/46,XY,add(11)(q13)	RAEB-1/46,XY,der(11)t(3;11)(q13.2;q13) [15]/46,XY [5]	No intervening marrows pre-transplant	Negative /72.5 /derivative 11 in 2 of 6 male (recipient) metaphases	Negative /70.7 derivative 11 in 7 of 12 male metaphases	Negative /12.4 /derivative 11 in 2 of 2 male metaphases	Negative /13.2 /derivative 11 in 4 of 4 male metaphases and 2 with additional t(1;7;13)(p31;q22;q13)	Negative /42.7 /deritivative 11 in 9 of 9 male metaphases with and 2 with additional t(1;7;13)	**RAEB-1 /68.3 /chi 46,XY,der(11)t(3;11)(q13.2;q13)[14]/46,idem,t(1;7;13)(p31;q22;q13)[3]/46,XX[3]**				Unknown/ Unknown	Clonal evolution
Days		−244	−15		21	66	170	352	546	658					
61	58 /INT-2	RAEB-2/Normal karyotype	Same specimen as original MDS diagnosis	Slightly hypocellular marrow with no dysplasia and no increase in blasts/normal karyotype	Negative /41 /not performed	Negative /0 /not performed	Indeterminate /0 /Neg by karyotype	Negative /0 /Neg by karyotype	**RAEB-2 /48 /46,XY[15]//46,XY[5] chimerism based on G-band polymorphisms**					Deceased of metastatic urothelial carcinoma with MDS /azacitidine	No clonal abnormality pre-transplant disease and relapse
Days		−106		−14	20	101	178	392	722					840 /118 (post-relapse)	
66	61 /unknown	RAEB-2/46,X,idic(X)(q13)[17]46,XX [3]	RAEB-2/46,X,del(X)(q22q26) [2]/46,X,t(X;10;3)(q26;q21;q12) [2]/46,XX with nonclonal abnormalities [2]/46,XX [4]	Hypocellular marrow with slight dysgranulopoiesis and 4% blasts/46,X,idic(X)(a13) [16]/46,XX [4]	Negative /5.1 /Neg by karyotype	Indeterminate ^e^ /0 /Negative by karyotype	**AML /60.9 /45,XX,-7[2]/46,idem,+21[8]/46,XX[10]**							Deceased of infection and persistent disease/ withdrawal of immunosuppression	Different abnormal clone
Days		−243	−107	−30	21	152	364							404 /40 (post-relapse)	
67	60 /High	MDS-U/unknown	MDS-U with <5% marrow blasts/47,XY,+8,inv.(12)(p13q13) [7]/46,XY [13]	No intervening marrows pre-transplant	Negative /4.8 /Neg by karyotype	Negative /2 /Neg by FISH	Indeterminate /0 /Neg by karyotype	Negative /0 /not performed	Negative /4.4 /Neg by karyotype	**RAEB-1 /91.8 /47,XY,+8,t(8;17)(q24.1;p13),inv.(12)(p13q13)[20]**				Deceased/ Haplo NK with IL-2 and Transplant	Clonal evolution
Days		−84	−15		7	57	88	172	252	310				632 /322 (post-relapse)	
74^b^	59 /INT-2	RAEB-1/45,XY,-7[19]/46,XY [1]	Same specimen as original MDS diagnosis	Normocellular marrow with no increase in blasts/Negative by FISH	Negative /0 /Neg by karyotype	Negative /3 /Neg by FISH	Negative /0 /Neg by FISH	Negative /0 /Neg by FISH	Negative /0 /Neg by FISH and karyotype	**AML /not performed /45,XY,-7[14]/46,XY[6]**				Deceased/ Hospice	Same abnormal clone
Days		−161		−27	20	99	127	192	358	583				583	
75^b^	58/ INT-2	T-MDS/normal karyotype	T-MDS with >10% marrow blasts/normal karyotype	Slightly hypocellular marrow with minimal dysplasia and no increase in blasts/unknown	Negative /0 /Not performed	Negative /0 /not performed	**Early relapsed myeloid neoplasm with slight trilineage dyspoiesis, borderline increased marrow blasts, and rare circulating blasts** ^**f**^ **/9 /46,XX[20]**							Deceased in hospice with progression to AML/ Immunosuppression withdrawal	No clonal abnormality pre-transplant disease and relapse
Days		−205	−130	−30	21	96	181							298 /117 (post-relapse)	
76	46 /INT-1	RAEB-2/46,XY,inv.(3)(q21q26.6) [6]/46,sl,+14,i(14)(q10) [3]/46,XY [11]	Same specimen as original MDS diagnosis	Slightly hypocellular marrow with mild dysplasia and no increase in blasts/normal karyotype	Indeterminate /6 /46,XY,inv.(3)(q21q26.2) [1]/46,XY [18].nuc ish(EVI1x2)(5’EVI1 sep 3’EVI1x1)[4/800]	Negative /6 /Neg by FISH and karyotype	Negative /2 /Negative by FISH and normal karyotype	Negative /7 /Negative by FISH and normal karyotype	Negative /0 /Neg by FISH	Negative (graft failure) /8 /Neg by FISH	Negative /8 /Neg by FISH	Negative /4 /not performed	**Relapsed MDS with dysplastic granulocytes and megakaryocytes, 4% to 7% blasts, and rare circulating blasts /68 /46,XY,inv.(3)(q21q26.2)[9]/46,XY[11]**	Alive in complete remission with GVHD/ Stem cell boost for graft failure (after 328 day marrow); second allogeneic sibling transplant for relapse	Same abnormal clone
Days		−102		−34	20	65	132	206	311	328	336	388	462	1001 /539 (post-relapse)	
80^c^	55 /INT-1	MDS associated with myelofibrosis/46,XX, t(3;8)(q26.2;q24.1) [17]/46,XX [3]	MDS associated with myelofibrosis ^g^/46,XX,t(3;8)(q26.2;q24.1) [3]/46,XX [17]	Hypocellular marrow with slight dysgranulopoiesis and borderline increased blasts/not performed	Negative /0 /Neg by FISH	Negative /0 /Neg by FISH	Negative /0 /Neg by FISH	**RAEB-2 /45 /46,XX,t(3;8)(q26.2;q24.1),del(5)(q11.2),del(11)(p11.2p15),der(12)t(5;12)(q11.2;p11.2)[cp7]/46,XX[12]**						Deceased of disease/ ALT-803 trial	Clonal evolution
Days		−375	−166	−18	21	100	183	370						436 /66 (post-relapse)	

RELAPSE MARROWS in BOLD

*Abbreviations*: *IPSS* International prognostic scoring system, *MDS* myelodysplastic syndrome, *CG* cytogenetics, *INT-1* Intermediate-1, *INT-2* Intermediate-2, *RCMD* refractory cytopenias with multilineage dysplasia, *RAEB* refractory anemia with excess blasts, *MDS-U* myelodysplastic syndrome, unclassifiable, *AML* acute myeloid leukemia, *T-MDS* therapy related myelodysplastic syndrome, *FISH* fluorescence in-situ hybridization, *DLI* donor lymphocyte infusion, *GCLAC* G-CSF priming, clorarabine, and high dose cytarabine, *ALT-803 trial* IL-15 Superagonist Clinical Trial, *GVHD* graft versus host disease, *LYF73636* clinical trial

^a^ Engraftment is reported as % recipient

^b^ Patient has a history of cytotoxic chemotherapy

^c^ Received myeloablative transplant

^d^ Days are relative to transplant date unless otherwise noted

^e^ Case 33 was called positive on study review due to identification of rare blasts with Auer rods; Case 66 was called positive on study review due to trilineage dysplasia including abnormal lobation of granuloccytes, small and hypolobated megakaryocytes, and ring sideroblasts

^f^ Progressed to AML 2 months after with only withdrawal of immunosuppression in intervening time

^g^ Institutional review reports as MDS associated with myelofibrosis versus RAEB-1

For this study, we retrospectively reviewed, blinded to the original interpretation and time-point, slides from the bone marrow biopsy immediately prior to the relapse specimen. The morphologic conclusion at the time of original interpretation and at the time of review for this study varied for nine of the cases, mainly between negative and indeterminate (six cases). Three cases (cases 81, 66, and 33) were re-interpreted as positive for myeloid neoplasm. Case 81 had dysplastic granulocytes, rare ring sideroblasts, atypical monocytes, and increased blasts. Case 66 showed trilineage dysplasia including abnormal lobation in granulocytes, small and hypolobate megakaryocytes, and ring sideroblasts. Case 33 lacked dysplasia and did not have an increase in blasts; however rare blasts with Auer rods were seen in the peripheral blood and bone marrow aspirate slides.

### Outcomes post relapse

Four of the five patients with early relapse and known follow-up were deceased, with a median survival of 306 days post-transplant (range 232 to 465 days) and 186 days post-relapse (range 58 to 367 days). The one patient (Case 79) alive at last follow-up of 625 days post-transplant/535 days post-relapse was treated for relapse with withdrawal of immunosuppression and donor lymphocyte infusion and achieved complete remission for approximately 2 years followed by relapse as AML for which she is undergoing treatment.

Eight of the ten patients with late relapse and known follow-up were deceased, with a median survival of 608 days post-transplant (range 298–2604 days) and 120 days post-relapse (range 40–583 days). Treatment protocols following disease relapse varied and are detailed in Table 3. Two patients were alive at last follow-up, 539 and 1892 days post-relapse, both in complete remission. Case 33 was 46 years old at the time of transplant and relapsed with AML for which she was treated with induction chemotherapy followed by HaploNK therapy with transplant. Case 76, also 46 years old at the time of transplant, had graft failure within a year of transplant for which he received a stem cell boost; subsequent relapsed disease (characterized by dyspoiesis in two lineages and 4% to 7% blasts) was treated with a second allogeneic sibling transplant.

## Discussion

Our study highlights the challenges of predicting MDS relapse post allogeneic HCT. Our patient cohort showed varied morphologic findings between pre-transplant disease and relapse with a large percentage of patients relapsing with a higher grade/higher blast MDS or frank AML. We found the presence of pre-transplant megakaryocyte dysplasia to correspond with the presence of megakaryocyte dysplasia in the relapse specimen but no consistency with erythroid or granulocytic dysplasia between pre- and post-transplant specimens. Similar to previous studies [11], cytogenetic abnormalities in the pre-transplant and relapse disease showed variability, including substantial proportions with clonal evolution pre-transplant to relapse or emergence of a previously undetected/undetectable clone. While the available flow cytometric data was too sparse in our cohort, studies on the immunophenotype of neoplastic blasts and maturing myeloid lineage cells in MDS pre-transplant and at relapse using modern and reproducible parameters (such as outlined by the European LeukemiaNet Working Group [12]) is needed. Our data highlight the need for additional more objective and quantitative measures of disease such as genetic profiling for common MDS mutations [13–15] for assessment of efficacy of interventions for relapse/impending relapse, such as that presented by Woo et al. [16].

Previous work by our group focusing on patients transplanted for MDS evaluated features of the marrow in the immediate pre-transplant biopsy, including blast percentage and the percentage of cytogenetically abnormal cells, and correlated with outcomes (survival and relapse). The Trottier study [17] evaluated patients from 1995 to the end of 2012 and only included those patients with abnormal cytogenetics in the diagnostic MDS sample. In contrast, in our current study, we performed a detailed retrospective evaluation of blood and marrow features prior to any preparative or cytoreductive therapy performed in anticipation of transplant and contrasted to blood and marrow features of the relapse specimen. In addition, our study includes only those patients with known relapse and we include all relapse patients and not those only with abnormal cytogenetics at MDS diagnosis. In reviewing the cases in common, 15 of the cases in the current analysis are in common with the Trottier report, which included 82 MDS patients. The current work expands on prior work in an attempt to identify features of impending relapse.

Our relapse patterns were consistent with the literature that describes the majority of relapses after allogeneic transplant for MDS occurring within the first year post transplant. Thirteen (62%) of the relapses in our cohort occurred within a year of transplant. There was a near even division of our patient cohort between those that relapsed before and after 6 months, defined as early and late relapse for this study. We found no specific features that differed between patients with late versus early relapse, with the exception of age and marrow blast percentage, likely correlating with myeloablative conditioning. Perhaps counter-intuitive to the usual poor prognosis attributed to therapy-related myeloid neoplasms, all three t-MDS cases were late relapses occurring 6, 19, and 49 months after transplant. Eighteen months has been used previously in the literature to define late versus early relapse [11]. In our cohort, the five patients who relapsed more than 18 months after transplant (patients 10, 17, 39, 61, and 74) did not show distinguishing features.

Relapse and impending relapse are difficult to define in the context of post-allogeneic stem cell transplant for MDS, particularly for low-grade MDS. Relapsed MDS does not always have increased blasts and dysplasia alone may be the only feature suggesting impending relapse. Unfortunately, the specificity of dysplasia in the post-transplant setting is low, even with published criteria for the definition and enumeration of ring sideroblasts and morphologic dysplasia [18, 19]. Dyspoiesis is described following bone marrow transplant in the erythroid lineage (including ring sideroblasts), granulocytic lineage, and megakaryocytes [20–22]. Granulocytic dysplasia can be seen in the context of immunosuppressive medication such as tacrolimus or mycophenylate mofetil [23]. Borderline increased blasts can also be seen with recombinant growth factor therapy (G-CSF and GM-CSF) administration and early robust marrow regeneration. In addition, a graft-versus leukemia effect may suppress persistent disease/impending relapse without additional intervention. Due to these factors, there is variability in the interpretation and clinical significance assigned to morphologic dysplasia in the post-transplant context. Our morphologic re-review of pre-relapse biopsies emphasizes the inter-observer variability in interpretation of dysplasia, with a different morphologic conclusion from the original interpretation in nine cases. Thus additional criteria beyond morphologic dysplasia are needed to help identify impending relapse.

Cytogenetic analysis is a valuable tool for detecting impending relapse. However, as a subset of MDS lack a cytogenetic abnormality and sampling may be a source of false negatives, results of cytogenetic analysis do not always yield the final answer. In addition, the relapse clonal abnormality may vary from the pre-transplant disease limiting the utility of directed FISH analysis. Genetic mutation analysis holds promise in detection of impending relapse, even in cases without cytogenetic abnormalities, although sampling and limits of detection are potentials for false-negatives and clonal heterogeneity within an MDS [24, 25] can create complexity in interpretation. Engraftment/chimerism studies can aid in identification of impending relapse; however, donor-cell derived MDS after allogeneic stem cell transplant [26] is a well described entity.

## Conclusions

A pragmatic approach to detection of MDS relapse and more importantly impending relapse following transplant is needed. Such an approach includes incorporation of morphologic, cytogenetic, and molecular data (including engraftment/chimerism studies). As with the initial diagnosis of MDS [27], it may take multiple sequential biopsies to make a definitive diagnosis of relapsed MDS. Detection of impending relapse is more difficult in cases with normal cytogenetics as morphologic features of dysplasia overlap with post-transplant changes and the dysplastic lineage(s) of the relapse disease may not correspond to those of the pre-transplant disease; molecular mutational analysis may prove very beneficial in these cases. While the appropriate intervention(s) for relapse and impending relapse is not well established, identifying definitive impending relapse and early relapse may allow for targeted interventions that may prevent a full blown relapse and improve patient survival. The pathologist’s role in diagnosis of impending relapse/relapse includes not only accurate morphologic identification of dysplasia and blast percentage, but an appreciation and knowledge of the multimodal approach to an MDS relapse diagnosis.

## Abbreviations

AML: Acute myeloid leukemia
HCT: Hematopoietic cell transplant
IPSS: International prognostic scoring system
MDS: Myelodysplastic syndrome
RAEB: Refractory anemia with excess blasts
WHO: World Health Organization
